# The Effect of Floral Resources on Parasitoid and Host Longevity: Prospects for Conservation Biological Control in Strawberries

**DOI:** 10.1673/031.013.10401

**Published:** 2013-10-06

**Authors:** Lene Sigsgaard, Cathrine Betzer, Cyril Naulin, Jørgen Eilenberg, Annie Enkegaard, Kristian Kristensen

**Affiliations:** 1 University of Copenhagen, Department of Agriculture and Ecology, Zoology Group, Thorvaldsensvej 40, DK-1871 Frederiksberg C, Denmark; 2 Aarhus University, Faculty of Science and Technology, Department of Agroecology, Research Centre Flakkebjerg, DK-4200 Slagelse, Denmark; 3 Aarhus University, Faculty of Science and Technology, Department of Agroecology, Research Centre Foulum, Blichers Allé 20, Postboks 50, DK-8830 Tjele, Denmark

**Keywords:** *Acleris comariana*, buckwheat, borage, *Copidosoma aretas*, diet, dill, functional biodiversity, phacelia

## Abstract

The strawberry tortricid, *Acleris comariana* Lienig and Zeller (Lepidoptera: Tortricidae) is an important pest in Danish strawberry production. Its most common parasitoid is *Copidosoma* *aretas* (Walker) (Hymenoptera: Chalcidoidea: Encyrtidae). To identify selective flowering plants that could be used to increase functional biodiversity, the longevity of *C. aretas* and its host *A*. *comariana* was assessed on 5 flowering species: buckwheat, *Fagopyrum esculentum* Moench (Caryophyllales: Polygonaceae); borage, *Borago officinalis* L. (Boraginaceae); strawberry, *Fragaria x ananassa* Duchesne (Rosales: Rosaceae); phacelia, *Phacelia tanacetifolia* Bentham (Boraginaceae); and dill, *Anethum graveolens* L. (Apiales: Apiaceae). Dill was only tested with *C. aretas*. Sucrose and pollen served as positive controls, and pure water as a negative control. In a subsequent field experiment, *A. comariana* larval density was assessed at 1, 6, and 11 m distances from buckwheat flower strips in 3 fields. The proportion of field-collected larvae that were parasitized by *C. aretas* or fungi was assessed. Among the tested floral diets, buckwheat was superior for *C. aretas*, increasing its longevity by 1.4 times compared to water. Although buckwheat also increased longevity of *A. comariana*, its longevity and survival on buckwheat, borage, and strawberry was not significantly different, so buckwheat was chosen for field experiments. *A. comariana* densities in the 3 fields with sown buckwheat flower strips were 0.5, 4.0, and 8.3 larvae per m per row of strawberry respectively. Of the collected larvae, a total of 1%, 39%, and 65% were parasitized by *C. aretas*, respectively. The density of *A. comariana* and the proportion parasitized by *C. aretas* were highly significantly correlated. Distance from floral strips had no significant effect on either *A. comariana* larval density or on the proportion of individuals parasitized by *C. aretas*. Few other parasitoids emerged from collected larvae, and no larvae were infected by entomopathogenic fungi. Still, total *A. comariana* mortality was significantly affected by distance to flower strips, with the highest mortality near the flower strips. As no effect of buckwheat flower strips on *C. aretas* parasitism was found, the positive effect they had on *A. comariana* control stems from unknown mortality factors. As literature indicates that buckwheat for flower strips can augment a more complex suite of natural enemies, one such mortality factor could be a non-consumptive predator and/or parasitoid effect, but this requires further study. If confirmed, buckwheat may be utilized together with a selective food plant, once identified.

## Introduction

The strawberry tortricid, *Acleris comariana* Lienig and Zeller (Lepidoptera: Tortricidae) is widely distributed in Europe, North America, China, and Japan (Guerrieri and Noyes 2005). It is considered one of the most important pests in the production of strawberry, *Fragaria x ananassa* Duchesne (Rosales: Rosaceae) (Lindhard et al. 2003) in Denmark. The larvae feed on leaves and flowers of wild and cultivated strawberry and also on fruit trees and some species of the rosaceous family (*Malus* spp., *Pyrus* spp., *Potentilla palustris* (L.), and *Geum rivale* (Alford 2007). The common parasitoid of *A. comariana* in Denmark is the polyembryonic egg-larval parasitoid, *Copidosoma aretas* (Walker) (Hymenoptera: Chalcidoidea: Encyrtidae), which is also found in the UK (Turner 1968; Guerrieri and Noyes 2005).

In Europe, natural control by *Copidosoma* is likely to prevent populations of many moth species in agriculture and forestry to reach economically damaging status (Guerrieri and Noyes 2005). A high natural occurrence of *A*. *comariana* parasitized by *C. aretas* observed in Danish strawberry fields (L. Sigsgaard, personal observation) makes it a potential candidate for conservation biological control. Conservation biological control is a modification of the environment or existing practices to protect and enhance specific natural enemies or other organisms to reduce the effect of pests (Eilenberg et al. 2001). Flower strips sown in the field can be used to provide natural enemies better access to pollen and nectar (Thompson and Hagen 1999). Selective food plants (Baggen and Gurr 1998) that can be used to augment *C. aretas* in strawberry fields while not favoring *A. comariana* would be preferred, as enhancement of pest fitness via the same mechanisms as natural enemies is potentially disadvantageous tobiocontrol (Zhao et al. 1992; Baggen and Gurr 1998; Begum et al. 2006).

Thompson and Hagen (1999) reviewed the effects of natural plant foods on adult parasitic wasps. In most cases, parasitic wasps feed on nectars as well as pollens. This feeding normally increases both longevity and fecundity, as found for *Trichogramma* spp. (Zhang et al. 2004). However, in predominantly proovigenic species, food resources such as flower strips may not result in higher fecundity, but in increased longevity, which indirectly may lead to increased parasitism as the time to encounter and parasitize hosts is increased (Thompson and Hagen 1999; Witting-Bissinger et al. 2008).

Pure proovigeny appears to be rare (Jervis et al. 2001), but an unpublished study by J.A. Harvey lists *Copidosoma floridanum* as proovigenic (Jervis et al. 2001). It is possible that *Copidosoma* spp. could be less reliant on adult food for fecundity, but fecundity of another *Copidosoma, C. koehleri*, increased when fed honey, and its longevity was increased when provided flowers to feed (Baggen and Gurr 1998).

No previous studies of floral diets exist for *C. aretas*, but buckwheat, *Fagopyrum esculentum* Moench (Caryophyllales: Polygonaceae), and borage, *Bor ago officinalis* L. (Boraginaceae), were documented to be of high value to another species from the same genus, *C. koehleri* (Baggen and Gurr 1998). Borage benefitted *C. koehleri* but not the host, the potato tuber moth, *Phthorimaea operculella* (Zell.) (Baggen and Gurr 1998). Likewise, phacelia benefitted *C. koehleri* but not its host (Baggen et al. 1999). Buckwheat and phacelia provided a superior diet to *Diadegma semiclausum* (Hellen), of higher value to it than to its host *Plutella xylostelia* (L.) (Winkler et al.2004; Lavandero et al. 2005), so these two plants could be considered selective for the parasitoid (Lavandero et al. 2005). The value of buckwheat flower strips has been demonstrated in apple orchards, where inter-sowing buckwheat increased parasitism by *Dolichogenidea* *tasmanica* Cameron on the lightbrown apple moth, *Epiphyas postvittana* (Walker) (Irvin et al. 2006). Tylianakis et al. (2004) found increasing levels of aphid parasitism with shorter distance to buckwheat plantings.

In the present study, the dietary value of flowers on the longevity *C. aretas* and its host *A*. *comariana* was assessed to identify selective food plants (Baggen and Gurr 1998) that could be used to augment *C. aretas* in strawberry fields while not favoring it's host, *A*. *comariana*.

## Materials and Methods

### Plants, pollen and sucrose

The longevity of *A. comariana* and *C. aretas* on diets of buckwheat, borage, phacelia (*Phacelia* *tanacetifolia* Bentham), and strawberry was tested. Pollen and sucrose (20% solution) served as positive controls, being a measure of longevity on either source of diet unaffected by flower architecture, sugar composition, or access to pollen and nectar, factors that may affect longevity (Lee and Heimpel 2003). Water was a negative control. The pollen was fresh bee pollen from Rosaceae (Percie du Sert (Rock Rose), www.pollenergie.fr). In addition, *C. aretas* longevity on a diet of dill, *Anethum graveolens* L. (Apiales: Apiaceae), was tested. A loss of dill plants designated for a longevity trial with *A. comariana* meant that this trial was not completed.

Plants were chosen based on a literature search identifying plant species with published value to parasitoids. Agronomic performance also was considered (Bowie et al. 1995). Buckwheat and phacelia are sucrose rich (Baker and Baker 1983; Irvin et al. 2007; Tompkins et al. 2010). Borage has a balanced composition and a high nectar production (Wykes 1953; Irvin et al. 2007), and strawberry and dill are glucose-fructose rich (Grünefeld et al. 1989; Abrol 1992; Irvin et al. 2007).

Buckwheat, borage, dill, and phacelia were grown from seeds. They were sown in sowing trays with 0–35 mm light sphagnum supplied with clay granulate, lime, and an average level of fertilizer, pH 6.0 adjusted with lime (Pindstrup 2, www.pindstrup.com). Seedlings were transplanted after 1 week to 13 cm pots with 10–30 mm light sphagnum with coarser structure, a high air content, supplied with clay granulate, an average level of fertilizer, pH 6.0 adjusted with lime (Pindstrup 4), and kept in the greenhouse at normal daylight and temperatures at around 20–22° C. Seedlings were kept in a greenhouse for the first 14 days at normal daylight and temperatures, around 20–22° C, before being transferred to outdoor netted cages (3 × 1.5 × 1.5 m). A staggered sowing ensured fresh flowers during the course of the experiment. Strawberry flowers were obtained from potted plants of the everbearing variety Ostara, which were grown in 3.5 L pots with the same fertilizer (Pindstrup 4). All plants were fertilized with irrigation water for a pH of 5.5 and a conductivity of 1.9. Strawberry plants were regularly pruned of developing fruits during the trials to ensure continued flowering.

### Insect rearing

*A. comariana* larvae (1^st^ through 5^th^ instar) were field collected in summer from 10 different commercial strawberry fields, both organic and conventional, in Zealand, Denmark, and reared individually in 30 mL plastic containers with ventilated lids. A strawberry leaf inserted in water-agar (1%) at the base of the container served as diet. The water-agar provided support and moisture to the leaf. Every second day, larvae received a fresh leaf and if necessary they were moved to a new plastic container. When a larva had pupated, it was transferred to an empty container. Pupae were checked daily for emergence of adults. Rearing took placed in a 20° C climate chamber (± 1° C) with a 16:8 L:D photoperiod. Newly emerged adults (0–24 hours old) were used in experiments. Sex is difficult to observe on live individuals, so both males and females were used, and sex was determined at the end of the experiments (Vandeurs 1956).

*C. aretas* overwinters as eggs in *A. comariana* eggs. Adult *C. aretas* were obtained from field collected *A. comariana* larvae. After the fifth instar, parasitoid pupae form inside the skin of dead larvae. Individual dead parasitized larvae with parasitoid pupae were transferred to empty containers (similar to the above except without water-agar and without ventilation holes) and observed daily for emergence. Newly emerged females (0–24 hr) were used in experiments. The sex of the live 1.0–1.2 mm long females was determined by antennal shape (Guerrieri and Noyes 2005), allowing the use of only females.

### Longevity of *A. comariana*

*A. comariana* longevity was assessed in plastic containers (7 cm diameter, 9.5 cm height) with a 2 cm diameter ventilation hole covered with filter paper in the top. Distilled water was provided in an open Eppendorf vial plugged with a folded piece of filter paper, serving as a wick extruding out of the vial, and ensuring a constant source of water. Flowers were provided in 30 mL plastic containers (3. 5 × 4 cm), with the stems inserted in 1% water-agarin the base of the container to sustain the turgidity and freshness of the flowers. Pollen grains were provided on a small, inverted, plastic lid (diameter 3.8 cm, lip 0.4 cm) to prevent contact with water. Sucrose was provided in the same way. Distilled water served as a control treatment. In each container, 1 newly emerged *A. comariana* was released. Fresh diet and water were provided daily in the morning to secure freshness and surplus of diet. Flowers have highest nectar availability in the morning (Lee and Heimpel 2003). Excised plants were used for trials since there is little evidence that nectar content and production of nectar are altered in excised plants (Wade and Wratten 2007). Adults were assigned to treatments distributing the staggered emergence over treatments. As sex was not determined before the death of an individual *A. comariana*, it was not possible to distribute treatments evenly between sexes. The number of replicates depended on the availability of fresh flowers. The higher number of replicates with buckwheat and borage reflect that these two flower species had a continuous high production of flowers.

### Longevity of *C. aretas*

The containers used for trials with *C. aretas* were the same 30 mL containers as used for rearing. Several small pinholes in the containers provided ventilation and were necessary to avoid condensation. The containers were inverted for use. Flower stems were fit through a hole in the lid and secured with parafilm. Outside the container, the stem was inserted in a similar container filled with about 20 mL 1% water-agar, providing the flower with water and support. Distilled water was provided on a 1 × 1 cm piece of filter paper in all treatments, and food (pollen grains or sucrose) was provided on a 1 × 1 cm piece of filter paper. Distilled water served as a control treatment. In each container, 3 newly emerged adult femalesfrom the same brood (i.e., identical siblings) were released. Fresh diet and water were provided daily to secure freshness and surplus of diet. Adults were assigned to treatments distributing the staggered emergence over treatments.

### Flower strips

Flower strips of buckwheat (1 m width, at least 40 m length) were established inside 3 different strawberry fields (var. Honeoye) at three conventional farms located in Zealand, where *A. comariana* had been found the 2 previous years. Farm 1 was located near Ringsted (55° 28′ 34″ N, 11° 49′ 51″ E), Farm 2 was near Klippinge (55° 21′ 24″ N, 12° 18′ 45″ E), and Farm 3 was near Skaælskør (55°, 16′ 27″ N, 11° 19′ 48″ E). No insecticides were used in the fields. Farms 1 and 2 did not use insecticides in strawberry, while Farm 3 often applied a pyrethroid treatment in strawberry in spring to control *Anthonomus rubi* and/or *A. comariana*. Strawberry was planted in double rows with 4 plants per m, with interrow distances of 60 cm in the double row and 100 cm between double rows. A split sowing of half the width of the buckwheat strip between 20–23 April 2011 and again 2 weeks later ensured flowering from early June until mid-August or later, providing flowering buckwheat throughout the period when *A. comariana* summer eggs and larvae were present. Flower strips were replacing strawberry rows, which were planted in the fields’1 longitudinal direction.

In Farm 1, the field was 120 m long from north to south and 50 m wide. The strip was placed in the center of the field. To the south and east there was a hedgerow, at least 30 m from the strip, to the west there was a cereal crop, and to the north there were farm buildings. In Farm 2, the field was 120 m from north to south and 50 m wide. This strip wassown the full length of the field and placed 10 m from a hedgerow to the east, separating it from another strawberry field. To the north, south, and west there were cereal fields. In Farm 3, the field was 120 m long from west to east and 50-80 m wide. The strip was 40 m long. To the south of the strip there was a cereal field separated from the strip by a grassy field margin with a few bushes. To the north there was a tarmac road and farm buildings, to the west strawberry tunnels, and to the east another strawberry field.

In all 3 fields there was a low presence of weeds. In Farm 1, the common flowering weeds were *Senecio vulgaris, Tripleurospermum* *perforatum, Veronica arvensis, Sonchus* *asper*, and *Stell aria media*. In Farm 2, the common weeds observed were *Tripleurospermum* *perforatum, Capsella bursapastoris*, *Carduus arvense, Rumex obtusifolius*, *Veronica persica*, and *Lamium purpureum*. In Farm 3, the common flowering weeds were *Senecio vulgaris, Crepis capilaris, Geraniaceae* *pyrenaicum*, and *Capsella bursapastoris*.

In spite of a very dry spring, buckwheat plants were able to establish. The density of *A. comariana* larvae was assessed visually in at least 30 1-m-stretches of strawberry plants in the field at 1 m, 6 m, and 11m distance from the flower strip once in late July (Table 2). In Farm 2, only the part of the field to the west of the strip facing away from the hedgerow was sampled. Field-collected larvae were reared as described above in a climate cabinet to determine parasitism and other mortality. Samples from different fields and distances were all kept and handled identically. Tortricid pupae from which adults had not emerged after 2 months were dissected. To reduce unknown mortality, dead larvae, which were expected to be killed by fungi (external symptoms), were placed individually in humid chambers (small plastic containers with moistened filter paper) at 20° C for at least 2 weeks to enhance fungal growth. Together with the information regarding parasitism, this created the basis for assessing the effect of natural enemies on mortality.

### Data analysis

Longevity was analyzed using mixed models (e.g., West et al. 2007). Before analysis, data were log transformed (log 10 (x + 1)) to meet the requirements for normal distribution and homogeneity of variances. The models used were reduced by removing non-significant higher order interactions (Bibby et al. 2004). Comparisons of individual diets were done by comparing the marginal means (least square means) using two-sided *t*-tests. The model used for *A. comariana* longevity included effect of diet and sex together with the interaction diet*sex, with the field where the larva were sampled as a random variable. For *C. aretas*, the model included the same fixed effects, with three random variables: 1) the field where the larva had been sampled, 2) the container (there were 3 *C. aretas* per container), and 3) the maternal descent of the *C. aretas* (on average 20–30 *C. aretas* emerged from a single dead larva, and in some cases such identical siblings were used in more than one container). Survival distributions for both species were analyzed with a non-parametric analysis using survival distribution plots and product-limit survival estimates (Kaplan and Meier 1958). Tests for homogeneity of survival periods over strata included the log-rank test, which is most sensitive to differences late in the survival periods, and the modified Wilcoxon test, which is most sensitive to differences early in the periods.

The effect of distance from flower strip on *A*. *comariana* larval density (log-transformed),proportion of collected larvae parasitized by *C. aretas*, proportion parasitized including other parasitoids, and proportion dead from unknown causes before becoming adult, as well as total mortality (no transformation needed), were analyzed using a mixed linear model with distance as a continuous variable and field as a random effect.

The parameters and tests for mixed models were performed using the mixed procedure of SAS (SAS Inst. 2008), while the nonparametric analyses were performed using the lifetest procedure of SAS.

## Results

### Longevity and survival of *A. comariana*

The highest longevity of *A. comariana* males and females, 35–39 d, was found on pure diets of sucrose and pollen, although the longevity was not significantly different from the longevity when fed borage and buckwheat (Table 1). Longevity of *A. comariana* (Table 1) was highly significantly affected by diet (F_6,238_ = 20.9, *p* < 0.0001) but not affected by sex (F_1,238_ = 1.7, *p* = 0.2), and there was no significant interaction effect of diet and sex (F_6,246_ = 1.3, *p* = 0.2).

**Figure 1.**
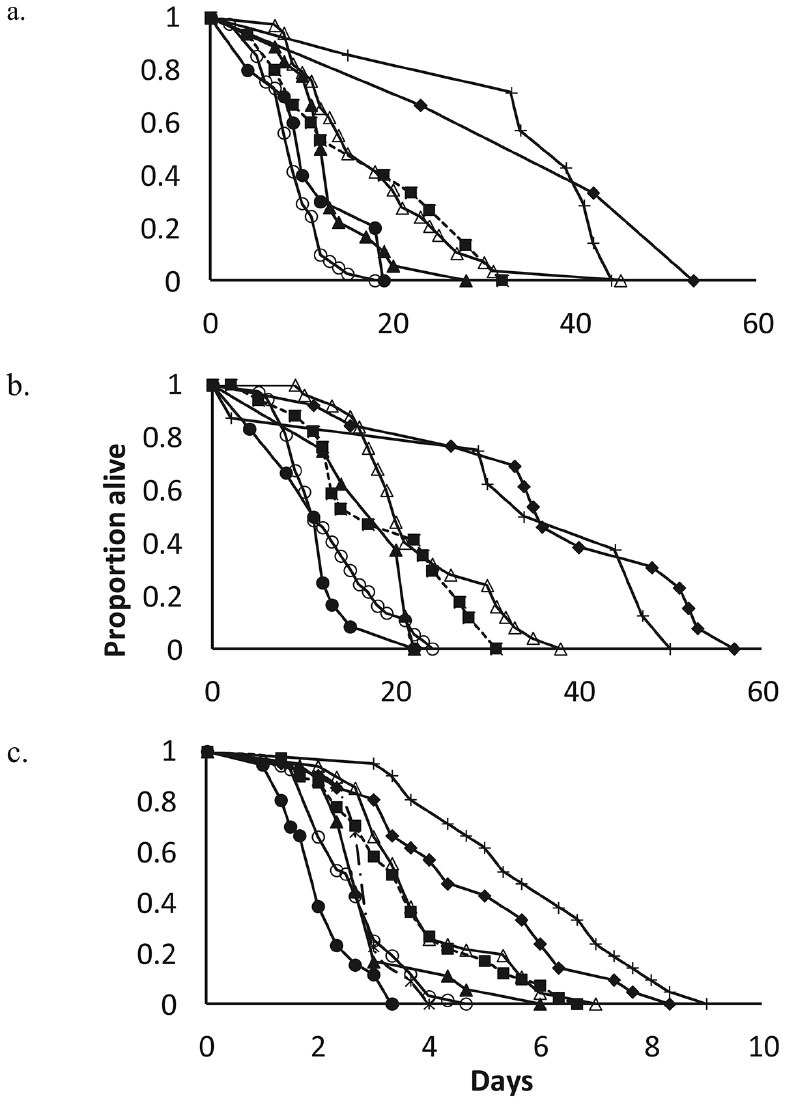
Survival of a) *Acleris comariana* **females, b) *Acleris*** *comariana* males, and c) *Copidosoma aretas* females on diets of buckwheat (Δ), borage (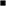), phacelia ( ), dill (--x--), and strawberry (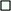), as well as on pollen (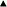), sucrose (+), and water control ( ). Survival on dill was only assessed for *C. retas*. High quality figures are available online.

As for mean longevity, survival distributions of females on borage, buckwheat, and strawberry (Figure 1a, b) were not significantly different early in the survival periods (Table 1). According to the survival analysis, phacelia was inferior to buckwheat, but not different from borage and buckwheat early in the survival periods. The survival analysis also showed significant difference between buckwheat and pollen late in the survival distribution and between borage and pollen throughout the survival distribution. For males, the value of pollen was superior to buckwheat. The value of floral diets for males was overall similar to that for females, though in contrast to females, early in the survival periods, strawberry was superior to water for males. A comparison of survival distributions of the 2 sexes showed no difference for a diet of phacelia, sucrose, or pollen. In 3 cases, survival of the 2 sexes differed: males lived longer than females on buckwheat and strawberry early in the survival period (Wilcoxon *p* < 0.01 and Wilcoxon *p* < 0.03 respectively), and throughout the survival period males lived longer on water (Wilcoxon *p* < 0.0001, Log-Rank, *p* < 0.0005).

### Longevity and survival of *C. aretas*

The highest longevity of *C. aretas* females on a floral diet was on buckwheat, with a mean of 3.8 days. On sucrose, longevity was a mean of 5.8 days (Table 1). The longevity of *C. aretas* was highly significantly affected by diet (F_7,229_ = 21.0, *p* < 0.0001). A comparison of population means showed that the highest longevity was obtained on a diet of sucrose, followed by pollen and buckwheat. The longevities on pollen and buckwheat were not significantly different from the longevity on borage, while the longevity on strawberry was equivalent to the longevity on dill. The shortest longevity of *C. aretas* was found on phacelia, being even shorter than the longevity on water (Table 1).

**Table 1.**
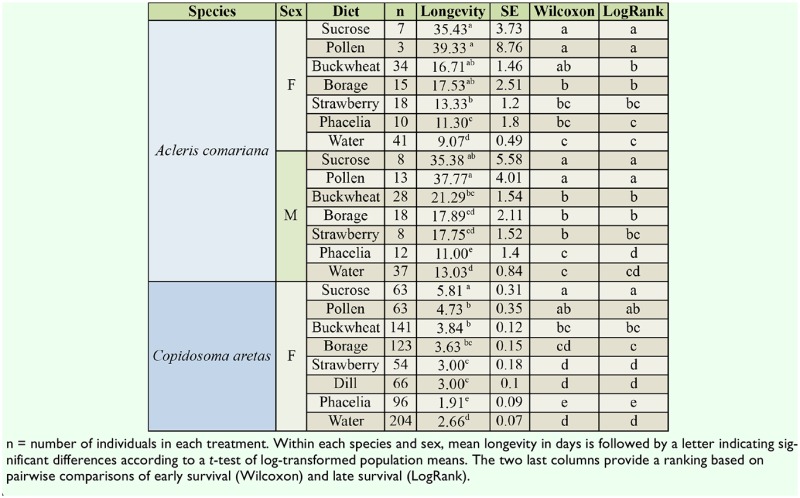
Mean longevity (± SE) of *Acleris comariana* and *Copidosoma aretas* on floral diets, pollen sucrose, and water.

The survival analysis showed similar trends. For *C. aretas*, the survival on buckwheat (Figure 1c) early in the survival period was not significantly different from pollen (Table 1). Buckwheat and borage survival periods were not significantly different. Buckwheat was superior to all other floral diets, while borage was not significantly different from dill early in the survival period. The survival distributions on strawberry and dill were not significantly different, but both were superior to the survival on phacelia. Survival on phacelia diet was inferior to survival on water.

### The effect of distance to flower strips on *A*. *comariana* density, parasitism, and mortality

A total of 663 *A. comariana* larvae were collected from strawberry plants at the 3 farms at distances of 1, 6, and 11m from the buckwheat strips (Table 2). At the time of sampling, larvae were 1^st^ through 3^rd^ instar, ensuring that the sample would represent the full parasitism by *C. aretas*, as it is an egglarval parasitoid, but would not represent total parasitism or hyperparasitism of all instars. Only 4 tortricids of species other than *A. comariana* were found, and those were excluded from the analysis. Of the collected *A. comariana*, 307 were parasitized by *C. aretas*.

**Table 2.**
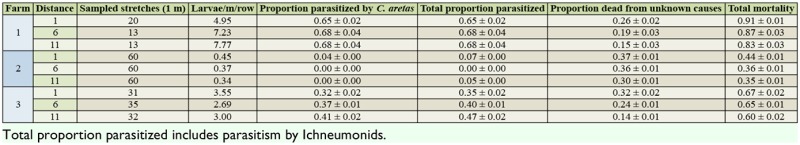
Density of *Acleris comariana* larvae, proportion of larvae parasitized by *Copidosoma aretas*, proportion dead from unknown causes before adult emergence, and total larval mortality in 3 strawberry fields at 3 distances from a flower strip of buckwheat sown inside each field.

Fifteen larvae were parasitized by Ichneumonids (2 in Farm 2, 13 in Farm 3). No hyperparasitoids emerged. 107 larvae and 47 pupae died of unknown causes. Dissection of dead pupae revealed no visible signs of parasitism. No mortality due to entomopathogenic fungi was found in dead larvae after incubation.

*A. comariana* densities in the 3 fields with flower strips were means of 6.7, 0.4, and 3.1 larvae/mrow respectively (Table 2). Across fields there was a highly significant correlation between the density of *A. comariana* and the proportion parasitized (N = 9, Pearson correlation coefficient = 0.98, *p* < 0.0001). There was no significant effect of distance from the flower strip on *A. comariana* density (F_1,5_ = 0.15, *p* = 0.72). Also, there was no significant effect of distance on the proportion of *A. comariana* parasitized by *C. aretas* (F_1,5_ = 1.6, *p* = 0.26). In all fields, mortality was highest near the flower strips (Table 2), with a significant effect of distance (F_1,5_ = 29.1, *p* = 0.003), and an estimated mortality by the flower strip (distance 0 m) of 34%, decreasing by 1.2% per meter distance to the strip (intercept ± SE: 0.34 ± 0.05, t = 7.1,*p* = 0.01; slope: -0.012 ± 0.002 , t = -5.4, *p* = 0.003). Likewise, total *A*. *comariana* mortality, including parasitism, was highest near the strip, with a highly significant effect of distance (F_1,5_ = 118.4, *p* < 0.0001) (intercept ± SE: 0.68 ± 0.14, t = 4.8,*p* = 0.04; slope: -0.008 ± 0.0011 , t = -6.7,*p* = 0.0011).

## Discussion

### Longevity and survival of *A. comariana*

Except for phacelia, which differed in dietary value from other floral diets but was still superior to water, the floral diets were equivalent in value for *A. comariana*. The longevity of *A. comariana* sexes as a function of diet was not significantly different, though the survival distribution analysis showed differences in 3 cases, as males survived longer on strawberry and buckwheat than females early in the survival period, and longer than females on water throughout the survival period.

The fact that *A. comariana* lived longer on sucrose than on any floral diet could be an effect of nectar accessibility. Moths are mostly inactive during the day, which means the nectar in the flowers has crystallized or become very gelatinous for the moths to feed upon by the time they become active (Traynier 1983). In the experiment with sucrose, the sucrose remained liquid and accessible.

The high longevity of *A. comariana* on pollen (not significantly different from that on sucrose) indicates that this species may utilize pollen. This has previously only been documented for Micropterigidae and the families Nympalidae (*Heliconius* spp.) (O'Brien et al. 2004; Wäckers et al. 2007) and Gelechiidae (*Phyllanthus* spp.) (Luo et al. 2011). Since pollen grains do not have to be broken in order for insects to extract nutrients from them, mechanical constraints should not limit pollen feeding (Jervis et al. 1993), but the use of bee pollen may overestimate the dietary value of pure pollen, as honey bees mix pollen with regurgitated nectar or honey for transport on their legs (Roulston and Cane 2000). The proportion of added nectar or honey has never been estimated directly (Roulston and Cane 2000), and it is possible that insects use sugars to aid the digestion of pollen (Roulston and Cane 2000).

The longevity of *A. comariana* females on the floral diets tested was not significantly different, except for on phacelia, which was equivalent to water. The low value of phacelia to *A. comariana* corresponds to the low value for it by another lepidopteran, *P. operculella*, in a study by Baggen et al (1999). Phacelia is sucrose rich, like buckwheat (Baker and Baker 1983; Irvin et al. 2007; Tompkins et al. 2010), therefore nectar composition alone does not explain its low value, while the long corolla of phacelia, outward pointing hairs on the style and ovary, and scale-like appendages within corollae, may limit nectar access, as discussed for *C. aretas* below.

The longevity found for *A. comariana*, ranging between 9 and 40 days depending on diet, corresponds with longevities reported for related species. *E. postvittana* had an average longevity of 14.5 days at 20° C (Gutierrez et al. 2010), and *Acleris glover ana* and *A. variana* males and females lived for about 14 and 28 days, respectively, in cages (EPPO 1980). In contrast to EPPO (1980), we did not find a difference in longevity between the sexes. The ability of male *A. comariana* to live longer when deprived of food may reflect a higher food requirement of females (Wäckers et al. 2007).

Lepidopterans typically produce viable eggs in the absence of adult feeding, but with age, adult diet can be an increasingly important source of egg carbon (O'Brien et al. 2004). As a consequence, a positive impact of feeding on lifetime fecundity can be due to a prolonged oviposition period or an increase in the daily fecundity when adult feeding increases the oviposition period (Wäckers et al. 2007). Further studies are needed on the ovipositional activity of *A. comariana*. If it is concentrated early in adult life, as was found for *P. xylostella* by Winkler et al (2009), an extension of the pest's longevity may not strongly affect pest abundance, whereas if the parasitoid has a prolonged oviposition period, an extension of longevity may lead to greater fecundity.

### Longevity and survival of *C. aretas* females

The highest longevity of *C. aretas* was found on pure diets of pollen or sucrose. Pollen can significantly increase the longevity of another genus of encyrtids, the *Trichogramma* spp. (Zhang et al. 2004). As for *A. comariana*, the use of bee pollen may have overestimated the dietary value of pure pollen. Among the floral diets, buckwheat was a superior diet for *C*. *aretas*, with an early survival distribution equivalent to that found on a pure pollen diet. Borage was also of high quality, while intermediate values were found for strawberry and dill, and longevity on phacelia was less than on water. The longevity of *C. aretas* on diets of the 2 sucrose rich flowers tested, buckwheat and phacelia, was very different, indicating that sucrose/hexose ratio cannot be used to explain parasitoid longevity (Tompkins et al. 2010), provided that insects can access the sugars of both flowers

Flower morphology can play an important role in parasitoids’ nectar searching and ability to extrude nectar (Wäckers 2004; Vattala et al. 2006), as the short mouthparts of parasitoids restrict feeding to exposed sugar sources, such as unspecialized flowers (Wäckers 2004), as found for *D. semiclausum* (Wäckers 2000; Winkler et al. 2006). Differences in corolla aperture do not seem to explain differences in dietary value. Dill and buckwheat both have exposed nectaries (Cawoy et al. 2008), and average corolla apertures of 2.2 and 6.6 mm, while borage has a corolla aperture of 0.10 mm (Baggen et al. 1999), strawberry has a wide corolla, and phacelia has a corolla aperture of 5.1 mm (Baggen et al. 1999). The long corolla of phacelia, outward pointing hairs on the style and ovary, and scale-like appendages within the corolla may limit nectar access by *C. aretas* (Baggen et al. 1999; Irvin et al. 2007) or even create stress, which may explain the low value that was found for this floral diet. Interestingly, it can be noted that phacelia did increase female longevity of another similar sized (0.9–1.1 mm long) congener, *C. koehleri* (Baggen et al. 1999; Guerrieri and Noyes 2005).

A higher parasitoid longevity when provided floral diet does not necessarily imply higher fecundity. Though *Copidosoma* spp. can be predominantly proovigenic (Jervis et al. 2001), increased longevity can indirectly lead to increased parasitism as the time to encounter and parasitize hosts is increased (Thompson and Hagen 1999; Witting-Bissinger et al. 2008). The fecundity of another *Copidosoma* species, *C. koehleri*, increased when fed honey, and longevity was increased when *C. koehleri* was provided with flowers to eat (Baggen and Gurr, 1998).

There are no other studies of *C. aretas* longevity. However, the longevity of *C. aretas* found in this study was shorter than the longevity of *C. koehleri*. In a laboratory trial at 25° C, *C. koehleri* lived on average 3.5 days without food or water, around 5 days with water or plants without flowers, 7 days with glucose, 10 days with coriander, 12 days with borage, and 13 days with dill (Baggen and Gurr 1998). In another study, the average longevity of *C. koehleri* with different floral diets was 7–17 days, and was longest with buckwheat (Baggen et al. 1998). *C. floridanum* longevity at temperatures from 15° C to 35° C varied from 30 days to 3 days, respectively, in the presence of food (20% levulose solution), but dropped to 8 days and 1 day, respectively, if only water was supplied (Stoner and Weeks 1974). Further studies are needed to clarify if other diets may provide longer longevity in *C. aretas*.

Among the flowers tested, it was not possible to single out a selective floral diet that was beneficiary to the parasitoid without at the same time also being beneficiary to the strawberry tortricid. However, buckwheat was superior for *C. aretas* while buckwheat, borage, and strawberry were not significantly different in quality for *A. comariana*. Therefore, buckwheat was selected for flower strip assays. Furthermore, as its seeds do not overwinter, it does not pose a potential weed problem the following year in strawberry, a problem that Danish strawberry growers have reported with dill. Other advantages of buckwheat are that it germinates easily, has a short sowing-flowering time, and seed is inexpensive and readily available (Bowie et al. 1995).

### The effect of distance to flower strips on *A*. *comariana* density, parasitism, and mortality

No effect of proximity to flower strips could be found on *A. comariana* density or parasitism. The incidence of parasitism by the ichneumonid was too low to draw any conclusions. Still, the high level of parasitism by *C. aretas* found in 2 of the fields, and the strong correlation between pest density and parasitism, shows the potential of this parasitoid to regulate *A. comariana*. A positive effect of proximity to a flower strip was demonstrated by Tylianakis et al. (2004), where aphid parasitism by *Aphidius rhopalosiphi* was 36% next to a buckwheat floral strip and exponentially declined to 0% at 14 m distance. Likewise, a distance effect on parasitism by *C. koehleri* of its host *P. operculella* in potato was found when assessed at distances from 1–20 m (Baggen and Gurr 1998). In that study, both species were field released prior to the analysis. Such augmented numbers were not used in our study, and relying on field populations may have reduced chances of demonstrating an effect. It is possible that greater distances than were used in our study may be necessary to discover the effects of foraging movements of *C. aretas* on resulting parasitism. Though *D. semiclausum* is about 4 times larger than *C*. *aretas*, it can be noted that Lavandero et al. (2005) found that it could easily move 80 m. To discover effects at such a scale would be difficult with normal field sizes, and a design where fields with or without flower strips are compared would be needed. Strawberry had completed flowering by mid-late June, before parasitism, but flowering weeds occurred in low density. If *C. aretas* were able to utilize weed flowers, this could have masked a distance effect of the floral strip. Finally, although buckwheat was found to increase the longevity of *C. aretas* under laboratory conditions, this does not mean that it will preferentially feed from buckwheat in the field. Witting et al. (2007) found that the relative attraction of trichogrammatids and 2 microhymenopteran parasitoids was not significantly higher in flowering buckwheat plots compared to plots where flowers had been removed or to non-flowering crabgrass controls.

A higher *A. comariana* mortality from unknown factors occurred near flower strips (34%), decreasing by 1.2% per meter distance to the strip, which shows that buckwheat had a positive effect on other mortality factors of *A*. *comariana* that was measurable at the scale studied. Unknown mortality may result from viral or fungal pathogens (Stairs 1966) or nonconsumptive predator effects, as stress responses induced by exposure to predator cues can increase the vulnerability of prey to other mortality factors, and mere exposure to predators can result in significant increases in mortality (McCauley et al 2011). Syrphid larvae often prey on *A. comariana* larvae inside the rolled strawberry leaves (L. Sigsgaard, personal observation). This occurrence was observed in this study. Syrphid adults are frequent visitors of buckwheat flowers (Cawoy et al. 2008). Other predators observed in strawberry include spiders, anthocorids, coccinellids, and carabids; however, their contributions to *A. comariana* mortality are unknown. A positive effect of flower strips with buckwheat on both predators and parasitoids was found in several other studies (Irvin et al. 2000; Nicholls et al. 2000; English-Loeb et al. 2003; Tylianakis et al. 2004; Eggenschwiler et al. 2010). Low fungal-caused mortality was also found in a 3-year survey of *A. comariana* mortality from parasitism and fungal pathogens, where fungal infection was only observed in 1 year and only in very few specimens (Sigsgaard et al. in press) This low mortality is noteworthy, since generalist insect pathogenic fungi are commonly found in soil and on insects in cropped fields (Meyling et al. 2011)

In conclusion, buckwheat was the best performing plant tested, but not a selective food plant. Although buckwheat is of high dietary value to *C. aretas*, and levels of parasitism in 2 of the fields tested were high, a positive effeet on parasitism could not be demonstrated at the distances tested in the field. A higher unknown mortality of larvae collected near flower strips was found, which calls for further study. Identical rearing conditions for all collected larvae exclude the possibility that this was an effect of rearing. As literature indicates that buckwheat for flower strips can augment a more complex suite of natural enemies, including predators (Irvin et al. 2000; Nicholls et al. 2000; English-Loeb et al. 2003; Tylianakis et al. 2004; Eggenschwiler et al. 2010), one such unknown mortality factor could be a non-consumptive predator and/or parasitoid effect (McCauley et al. 2011).

For conservation biological control of *A. comariana.*, a selective food plant for *C. aretas* remains to be identified. If a positive effect of buckwheat on *A. comariana* is confirmed, buckwheat augmenting a more complex suite of natural enemies of *A. comariana* may be utilized together with a selective food plant in flower strips, once it is identified.
